# Joint Consultation by a Neurogeneticist of Movement Disorders and a Psychodynamic Psychotherapist: An Occasion for Subjectivization

**DOI:** 10.3389/fpsyg.2020.01828

**Published:** 2020-08-05

**Authors:** Olivier Putois, Marie-Frédérique Bacqué, Mathieu Anheim

**Affiliations:** ^1^SuLiSoM UR 3071, Faculté de Psychologie, Université de Strasbourg, Strasbourg, France; ^2^Service de Psychiatrie, Santé Mentale et Addictologie, Hôpitaux Universitaires de Strasbourg, Strasbourg, France; ^3^Institut d’Immunologie et d’Hématologie, Institut Thématique Interdisciplinaire TRANSPLANTEX NG, Université de Strasbourg, Strasbourg, France; ^4^Service de Neurologie, Hôpitaux Universitaires de Strasbourg, Strasbourg, France; ^5^Institut de Génétique et de Biologie Moléculaire et Cellulaire (IGBMC), INSERM U964, CNRS UMR 7104, Université de Strasbourg, Strasbourg, France; ^6^Fédération de Médecine Translationnelle de Strasbourg (FMTS), Université de Strasbourg, Strasbourg, France

**Keywords:** subjectivization, therapeutic setting, consultation, neurogenetics, movement disorders, medical complaint, transference, psychotherapeutic processes

## Abstract

We set out to model a joint therapeutic setting meant to address both medical care and the transferential processes at stake in specialized neurogenetics consultations. Previous authors have explored joint consultation settings with a specialized physician and a psychodynamically oriented psychotherapist, however, few have attempted to provide a model of its transfero-countertransferential dynamics. We aim to do the latter by focusing on a subset of patients to whom such consultations are offered “on the spot.” We want to explore situations in which they initially deny the transference’s contribution to their complaint, when addressing it would instead prove to be beneficial to them, even medically speaking. Standard neurogenetics consultations put the physician in a double-bind position. These patients’ conflicting complaint both manifests transferential expectations and denies them by adhering to medical elements. Since the physician’s challenge is to avoid colluding with the patient’s denial, a joint setting would enable him to address the medical content of the patient’s complaint while simultaneously letting its transferential elements emerge, allowing for the psychotherapist to use them to induce subjective integration (subjectivization). We conceptualize this jointly induced subjectivization by drawing on Fain’s work on primary hystericization of the complaint (inspired by Freud’s late indications). We finish with an example of subjectivization of a family’s complaint based on an adolescent’s limb tremor, which had no genetic or neurological etiology. Its seemingly conversional nature appeared in light of her father’s reaction to our subjectivizing response: his latent transference was likely underlying his daughter’s symptom.

## Introduction

In 1957, Balint (1957) highlighted the role of transference in the patient’s complaint to the physician, and the subsequent role of the latter’s countertransference in proposing an adapted response (thereby addressing the placebo effect, cf. Roques et al., 2014). In his wake, Lacan has insisted that the physician must address patient transference aside his explicit medical demand (Lacan, 1966; cf. Potier and Putois, 2018).

In this light, physicians and psychodynamically oriented psychotherapists have devised diverse outpatient clinical settings in various medical contexts and specialties, in order to address the transferential dimension of patient complaint. These include dedicated liaison psychiatry consultations associated with somatic healthcare pathways (Consoli, 2010), psychoanalytic consultations within hospital services (Del Volgo, 1997), bifocal patient-tailored follow-ups (e.g., Missonnier and Boige, 1998), and joint consultations (e.g., Misery and Chastaing, 2003; Ullnik, 2007).

To our knowledge, all these settings share an initial step. They explicitly propose the patient to engage with a distinct psychical dimension of his complaint in a dedicated therapeutic setting. Therefore, all patients come to the consultation knowing that the physician believes that their complaint includes such a dimension.

Yet, many outpatients are not ready to acknowledge the presence of transference, even though it pervades all consultations. To the despair of many physicians, this subset of patients focus on the organic dimension to deny transference. They therefore maintain a splitting between soma and psyche, which sometimes bears on life-threatening medical matters (e.g., treatment interruption). This is especially true for chronic illnesses requiring specialized expertize regarding the body, as in neurogenetics; such patients wouldn’t spontaneously acknowledge this psychical dimension, let alone request to address it.

Such a defensive attitude warrants an additional step prior to referring these patients to a consultation explicitly dedicated to these transferential stakes. We need a setting helping them to access the implicit transference present in their complaint while at the same time responding to its explicit medical content.

This is the aim of our joint neurogenetics consultation: it is a therapeutic setting devised to help patients access this dimension (and enable subsequent psychotherapeutic work) within the context of addressing a medical complaint. This model also seeks to contribute to research on therapeutic settings facilitating psychodynamic processes.

### Denial and Transference in the Standard Outpatient Neurogenetics Consultation

#### The Standard Neurogenetics Consultation

Our consultation takes place in a neurology unit: it is proposed to a third of outpatients coming for a consultation in neurogenetics of movement disorders (MA directs the reference center). We start with standard (non-joint) consultations; MA’s perception of their psychical stakes was our starting point. Only de-identified data are used in this paper. An ethics approval was therefore not required for the use of this material as per the Institution’s guidelines and national regulations.

The medical aim of standard neurogenetics consultations is either to diagnose or to assess the evolution of a disease. The first consultation takes place within a pre-existing (and often long) care pathway. It welcomes patients referred by general practitioners or physicians in neighboring specialties. In inpatient consultations, a whole team including nurses, hospital care assistants, etc. provides care to the patients. Many doctors are residents or fellows, but not professors. In standard outpatient neurogenetics consultations, patients have a “renowned Professor” all to themselves. This narrower and more idealized transferential focus is crucial to our subsequent design of a joint consultation.

Movement disorders typical of pathologies encountered in this consultation aren’t always the salient element in the clinical picture. Such pathologies comprise ataxias, spastic paraplegias, Tourette’s, Parkinson’s, etc., and some patients are therefore affected with dementia.

Diagnosis always includes expert medical examinations, and sometimes genetic sequencing. In clinical examination, the physician focuses on the patient’s bodily signs, and discusses his current life context. This requires the patient to accomplish motor tasks to help the physician assess movement accuracy and its evolution over time. When asked to show how they control their body, some patients are reluctant and express shame for “What they are turning into.” Motor deterioration has a profound effect on body image. It is common to hear patients say things that echo one’s feeling of taking a driver’s test: they “don’t perform well under the stress, especially when they are being observed.” Clinically speaking, this is quite true, and highlights the complex interplay between bodily capacities and interpersonal emotional relations. It is therefore crucial for the physician, through verbal exchanges, to lighten the atmosphere in order help the patient relax so as to have a more accurate representation of his real-life motor skills.

The results of clinical examination, as well as the patient’s account of the life context, are also discussed with the accompanying caregiver(s) in the presence of the patient, especially in the context of dementia. Their accounts often diverge, sometimes considerably: situations of handicap are often a source of denial. This denial can have deleterious consequences, as when a patient ignores the side effects of levodopa (standard treatment for Parkinson’s), such as compulsive behavior like online betting.

Another feature of these consultations is the role of intergenerational transmission: firstly at the biological level of genome transmission, and secondly at the relational level of its effects on the guilt-laden relationships between family members (see, e.g., Gargiulo, 2018). Patients oftentimes come with a caregiver who almost immediately asks about information regarding his or her potential carrier status.

#### b. The Physician’s Double-Bind

The condition affecting such patients has a multifaceted psychological impact. Therefore, the patient’s psychical life gives a personal meaning to his experience of the condition (his “illness”): this personal meaning is expressed in the transferential elements within his complaint. These account for phenomena such as treatment interruption and omission of medically relevant information.

Yet in everyday practice, faced with transference, the physician frequently experiences that he is either:

1. Expected to address it directly as a psychotherapist, for which he is not trained and should therefore avoid in order not to overlook the medical complaint (even psychotherapy-trained physicians generally refer to a colleague, Missonnier and Boige, 1998);
2. Implicitly told by the patient that the latter is not interested in addressing this contribution, in spite of its effects on his condition or everyday life.

He is thus in a double-bind position (Bateson, 1972). In each case, he is expected to respond to the psychical dimension, while at the same time being prevented from doing so by the patient’s denial of either the psychical or somatic dimension. Trying to turn the medical consultation into a psychotherapeutic one denies that at that very moment, the physician (a specialist of the body) is in no institutional position and/or lacks the qualifications to undertake psychotherapy. Encountered situations present a frequent denial of transference. Trying to turn the consultation into a psychotherapy session is a partial denial since the patient could directly go to a psychotherapist.

A physician who senses this transferential dimension also feels that another side of the complaint denies it by focusing on somatic elements, viewed from a strictly medical perspective. The physician’s situation could be understood in light of what psychoanalysis highlights as the conflict, within patient complaint (”Klage,” cf. Freud, 1918), between transference and defense through denial. Transference refers to a search for psychotherapeutic help to alleviate mental suffering, while denial is a counter-attitude that denies the existence of a therapeutic object and therefore blocks transference. This conflict, which is internal to the complaint, manifests an underlying splitting of the Ego (see, e.g., Freud, 1918, 1937, 1938; Winnicott, 1955).

Experiencing this double-bind position, MA sought OP’s complementary expertise.

### 2. From Common Denial to Subjectivization

Expanding on Freud (1937), French psychoanalyst Fain (1971, 1980, 1982) commented that the analyst, when faced with this conflictual complaint, can either:

1. Respond directly to what the patient says he needs or wants (most often by giving real-life advice, etc.), thereby short-circuiting the possibility of addressing underlying transference;
2. Address the transference by hearing the complaint but responding to what it leaves aside, typically regarding the patient’s own contribution in what he complains about.

In the first, the analyst is like a mothering caregiver communicating to an infant that she is everything he needs and that he shouldn’t desire any alternate object (parent, etc.) since she does the same by focusing on him only. If the infant complies, “identification through a community of denial” (Fain, 1980, 1982) occurs. By drawing on the infant’s spontaneous tendency to regard her as his sole object, she induces infant identification. In a specialized medical consultation, the object of common denial is often transference itself.

Ignoring transference has important iatrogenic effects. Patients could either solicit further medical consultations and examinations, which would subsequently reinforce the initial denial and make it more difficult to later address transference. Or they could adopt alternative behavior tentatively soothing the distress which motivated transference in the first place. We witnessed several cases of unmentioned substance abuse (to soothe psychical suffering) in patients whose medical history started with such an initial denial.

How would one address the denied transferential elements latent within the complaint?

Freud laid out the aim of psychoanalysis as a specific form of psychotherapy: “Where Id was, there Ego shall be” (Freud, 1933, p. 79). This means: repressed (broadly speaking) elements should be integrated to the subject’s psychic life through work on transference. Since Cahn’s (1991) seminal work, French post-Freudian psychoanalytic tradition generally calls this aim subjectivization (”subjectivation,” e.g., Carel, 2006; Chabert, 2006; Kaës, 2006; Penot, 2006; Roussillon, 2006). Subjectivization has often been understood in terms of “subjective appropriation” (Roussillon, 2006).

Various works (Dejours, 1986, 2001, 2006; Roussillon, 1999) have followed Freud’s (1937, p. V) advice and emphasized that enabling subjectivization requires to acknowledge the splitting of the Ego expressed in the complaint. This has led authors such as Cahn (2002) to claim that the subjectivization of a complaint requires a prior regression to subjectalization (“subjectalization”), a transformative and more libidinally gratifying transferential re-enaction of the interactions with the mothering caregiver. The patient receives a primary narcissistic cathexis (holding, etc.) from the caregiver acting as a protective shield responding to her needs. By strengthening the split-off infantile part of the Ego, this subjectalization in turn enables subjectivization: the integration of the Id through an object-targeted type of transference.

We would like to contribute to the theory and technique of subjectivization by proposing a model of joint consultations where denied transferential expectations can be subjectivized insofar as the physician first lets the patient express his manifest complaint, laden with denied transference.

The originality of this model lies in its insistence on the expression of manifest complaint as a condition to subjectivization, which we define as primary hystericization of the complaint through a specific group dynamics.

To that effect, we expand on Fain’s notion of “censorship of the lover” (Fain, 1971; Braunschweig and Fain, 1975). It refers to the moment when a mothering caregiver, during infant holding and care, starts daydreaming about another object of desire (partner, etc.). In so doing, a shift happens in her mind: she becomes a lover and no longer a caregiver. This interrupts the exclusive cathexis of the infant through holding: she thus ceases to act as a protective shield (Freud, 1926). While frustrated not to remain her sole object of interest, the infant is also led to wonder what she was led to daydream about, and to share her desire for this other object. Thus introduced to triangularization, the infant experiences “primary hystericization” (“hystérization primaire,” Fain, 1971), which is the opposite of identification in the community of denial.

The physician’s position is similar to that of an analyst trying to elicit a movement of primary hystericization by communicating to a patient that the latter’s complaint leads him to daydream about something else (the patient’s unconscious transference). This type of conception captures the clinical advantages of a joint setting, wherein the more a physician engages with somatic cure (diagnosis and therapy, corresponding to maternal holding and care of the infant), the easier it is for the psychodynamic psychotherapist to address transference on the basis of the patient’s complaint.

In this setting, MA first asks patients if they’d accept OP’s presence in the consultation (95% out of about 100 agreed). Then, MA focuses on somatic care, like a substitute of the primary caregiver: patients focus on his questions. The comfort provided by his reassuring attitude leads them to express a complaint rich with denied elements. When relevant, OP responds to these elements by indicating that perhaps something else is also expected from the consultation: this indirectly refers to transference and makes the consultation explicitly psychotherapeutic, as well as medical. In other words, the two functions of mothering caregiver and daydreaming lover, both comprised in the infant’s experience of the primary object, are occupied by MA and OP (such functions vary depending on the situation). Our approach to the joint consultation thus differs from Ullnik (2007): his patients explicitly seek joint consultations, and he follows a semi-structured interview to gather material for subsequent psychotherapeutic interventions. Misery and Chastaing (2003) seem closer to our perspective even though they explicitly offer a joint consultation (while we propose OP’s presence on the spot, as an open possibility); also, they mostly focus on diagnosis. Moreover, they do not detail intervention techniques and psychical processes.

We close with an example. In pre-briefing, MA tells OP that a father has come with his 16-year-old daughter to receive the results of a targeted genetic sequencing because of her left leg tremor. This was decided after a preliminary interview on possible antecedents on both sides of the family – none were mentioned. The results are negative, which makes MA uncomfortable: it reinforces his clinical feeling of a “functional” symptom, even more so as the adolescent presents an indifferent attitude. Furthermore, the family context is complicated. The parents are in the process of getting a divorce and the accompanying father was forced to leave the house where his daughter and her mother live. MA proposes that OP participates; they agree spontaneously. MA explains that the results are negative and that, since other avenues have already been explored, the symptom is likely functional. Not much happens (no irritation or despair) besides their apparent detachment. MA quickly glances at OP. While MA took care of the adolescent’s somatic complaint, the negative results of genetic sequencing prevented a reinforcement of a father–daughter community of denial, which could have been strengthened by positive results.

OP mentally remarks the general lack of surprise. They do not seem to explicitly seek another response, yet they came. How could this seemingly hysterical symptom be approached from a different perspective?

Taking a moment to let a somewhat uncomfortable silence sink in while daydreaming, OP addresses the daughter and asks if “someone around her had something resembling what she has?” “Around” here referred to anyone, from friends to family. The goal was primary hystericization of underlying transference. What are they looking for? Are they as uninterested as they seem with medical results?

Since they told MA that her condition was without precedent in the family, OP expected that the response could be “a friend with a related condition,” or even a “forgotten” family member (omissions are frequent in genetics consultations). But after a pause, her father exclaims, “I had the same thing at the same age! No one found out what it was, and it disappeared shortly afterward.” The negative outcome of the medical response (sequencing) to the adolescent’s somatic complaint enabled OP to ask an open question eventually lifting the father’s repression and previous omission of his own adolescent tremor. Our joint response to his daughter’s somatic symptom enabled him to recognize in it an echo of his adolescence, and thereby prevent a community of denial. What father–daughter bond was her somatic symptom meant to express? Impossible to tell so far. OP remains silent. The father then says, “I will meditate upon what happened here today,” somewhat acknowledging that his daughter’s symptom expressed his own transferential need, and that our joint response opened an avenue to potentially explore it. Without MA’s response, OP’s question would not have had this type of effect; both caregiving and subjectivizing functions need to work jointly.

## Conclusion, An Occasion for Subjectivization

This situation illustrates the advantages of our setting. It would have been more difficult for MA to explicitly refer to a colleague for a separate psychotherapeutic consultation; doing so could have strengthened the father–daughter denial. This would also raise the question of the address: the father needed it at least as much as his daughter, but would a physician alone have addressed the whole family to a psychotherapist?

In contrast, the implicit interpretative effect following OP’s intervention, which remained neutral as to the cause (psychical or physical), was less confrontational than a direct psychotherapy referral. Also, the father’s denial could only be lifted by drawing on MA’s medical results in the same temporal sequence. Such a group sequence could be more unlikely with separate psychotherapeutic and medical consultations. This intermediate format, between specialized consultation and psychotherapeutic follow-up, is meant to help access the latter by broadening its indication range.

Our model aims to implement psychodynamic-oriented practices in medical specialities where seemingly strictly somatic symptoms can be used to reinforce a denial of the psychical. Since subjectivization draws on denied transference emerging in the initial medical exchange, we would hypothesize that in psychiatry, a single psychodynamically trained psychiatrist could produce the same type of effect, for example, by addressing transferential stakes in the context of a discussion regarding pharmacology, as in exchanges about patient’s resistance to treatment (Mintz and Belnap, 2011). As an expert of pharmacology and psychotherapy, he could subjectivize the medical complaint by shifting from the medical to the transferential level. Is this made possible by the nature of patient transference on the psychiatrist? After all, even when asking for medication, patients often want it to change psychical processes (e.g., mood alterations): he might therefore appear from the start as potentially addressing psychical issues. This is just a hypothesis.

## Author Contributions

OP took part in the consultations, designed the conceptualization, co-designed the clinical setting, and wrote the manuscript. M-FB advised OP during his research and gave indications during manuscript conception. MA initiated the project, took part in the consultations, co-designed the clinical setting, and participated in writing the manuscript. All authors agreed on the final manuscript.

## Conflict of Interest

The authors declare that the research was conducted in the absence of any commercial or financial relationships that could be construed as a potential conflict of interest.
